# Distinct Proteomic Profile of Spermatozoa from Men with Seminomatous and Non-Seminomatous Testicular Germ Cell Tumors

**DOI:** 10.3390/ijms21144817

**Published:** 2020-07-08

**Authors:** Manesh Kumar Panner Selvam, Marco G. Alves, Tânia R. Dias, Peter N. Pushparaj, Ashok Agarwal

**Affiliations:** 1 American Center for Reproductive Medicine, Cleveland Clinic, Cleveland, OH 44195, USA; pannerm@ccf.org (M.K.P.S.); taniadias89@gmail.com (T.R.D.); 2 Department of Microscopy, Laboratory of Cell Biology, Institute of Biomedical Sciences Abel Salazar and Unit for Multidisciplinary Research in Biomedicine (UMIB), University of Porto, 4050-313 Porto, Portugal; alvesmarc@gmail.com; 3 Faculty of Health Sciences, Universidade of Beira Interior, 6201-001 Covilhã, Portugal; 4 King Abdulaziz University, Center of Excellence in Genomic Medicine, Jeddah 21589, Saudi Arabia; peter.n.pushparaj@gmail.com; 5 Department of Medical Laboratory Technology, Faculty of Applied Medical Sciences, King Abdulaziz University, Jeddah 21577, Saudi Arabia

**Keywords:** seminomatous, non-seminomatous, sperm proteomics, male fertility, diagnosis

## Abstract

Testicular germ cell tumors (TGCTs) are predominant in young males (15–44 years). Seminomatous and non-seminomatous TGCTs account for about 98% of all TGCTs cases. In this study, we aimed to compare the sperm proteome of patients with seminomatous and non-seminomatous TGCTs to identify possible protein biomarkers that could help distinguish between them in a non-invasive manner. We analyzed semen samples from patients with seminomatous or non-seminomatous TGCTs (*n* = 15/group) that were cryopreserved before the start of cancer treatment. Quantitative proteomic analysis was conducted on pooled samples (*n* = 3/group) and a total of 258 differentially expressed proteins (DEPs) were identified. The overexpression of acrosin precursor (ACR) and chaperonin containing TCP1 subunit 6B (CCT6B) as well as the underexpression of S100 calcium-binding protein A9 (S100A9) in the spermatozoa of patients with non-seminomatous TGCTs were validated by western blotting conducted on individual samples (*n* = 6 for seminomatous group and *n* = 6 for non-seminomatous group). Our overall results suggest an association between the higher and faster invasiveness of non-seminomatous TGCTs and the altered protein expressions, providing important information for future studies.

## 1. Introduction

Testicular germ cells tumors (TGCTs) are a heterogeneous group of neoplasms occurring in the male germ cells [1,2]. Germ cells are essential for male reproduction as they differentiate into spermatozoa within the testis [3]. Although TGCTs are a rare type of tumor among men, they represent a major threat to male fertility. The main types of TGCTs are classified as seminomatous and non-seminomatous, which represent up to 98% of all TGCTs cases, while the remaining refer to spermatocytic tumors [4]. The prevalence of seminomatous and non-seminomatous TGCTs is similar, but some patients (15%) can also present both types [5]. Non-seminomatous TGCTs are considered more aggressive than seminomatous TGCTs because they grow and spread faster, and are also less sensitive to radiation treatment [6]. There are four sub-types of non-seminomatous TGCTs: embryonal carcinoma, teratoma, yolk sac tumor, and choriocarcinoma, which commonly occur in combination [7].

Although TGCTs have a survival rate of over 95%, their treatment highly affects patients’ fertility potential [8,9,10]. Sperm banking is recommended to patients with TGCTs prior to cancer treatment to increase their chances of having children afterward [11,12]. In fact, after the cancer therapy, the chance to establish a pregnancy by natural conception is 30% lower [13] and assisted reproductive technology (ART) may be required [12]. The diagnosis of TGCTs is mainly based on the histological analysis of a biopsy of the testicular mass that is classified according to the World Health Organization (WHO) criteria (e.g., tumor size, multiplicity, and extension) [14,15]. To support the diagnosis, several serum tumor markers such as α-fetoprotein (AFP) and/or human chorionic gonadotropin (HCG) are also analyzed [14].

Recently, proteomics has emerged as a valuable tool to investigate the molecular basis of health and disease [16]. Many studies have been focused on the analysis of sperm and seminal plasma proteome to understand their role in male reproductive function and associated diseases [17,18,19,20]. In previous studies, we compared the sperm proteome of healthy fertile men either with patients with seminomatous TGCTs [21] or non-seminomatous TGCTs [22]. These studies showed that the altered expression of several proteins involved in sperm function was responsible for the reduced fertility in men with TGCTs prior to cancer therapy when compared to proven fertile men. Hence, the identified proteins could be used as biomarkers for the diagnosis of subfertility/infertility in patients with TGCTs. However, there are still no sperm protein biomarkers that distinguish seminomatous from non-seminomatous TGCTs, which could be helpful in their non-invasive diagnosis. In this study, we aimed to compare the sperm proteome of patients with seminomatous and non-seminomatous TGCTs by liquid-chromatography tandem mass spectrometry (LC-MS/MS) and identify possible biomarkers for its distinct diagnosis.

## 2. Results

### 2.1. Semen Parameters Were Similar between Patients with Seminomatous and Non-Seminomatous TGCTs

There were no differences in the analyzed semen parameters when comparing seminomatous and non-seminomatous samples (Table 1). Besides, all the samples were considered normozoospermic according to the WHO 2010 criteria (WHO 2010).

### 2.2. Identification of the Differentially Expressed Proteins by LC-MS/MS

**Figure 1:**
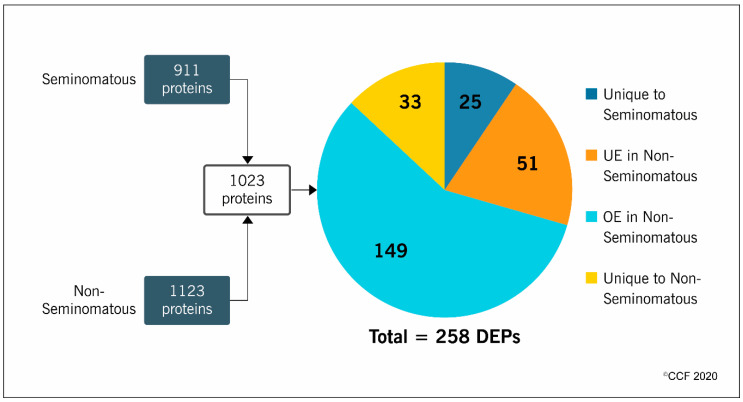
Number of proteins identified by proteomic analysis of spermatozoa samples obtained from patients with seminomatous and non-seminomatous testicular germ cell tumors, and expression profile of the differentially expressed proteins (DEPs) identified after comparative analysis between the experimental groups. OE, overexpressed; UE, underexpressed.

LC-MS/MS analysis identified 911 proteins in the seminomatous group and 1123 in the non-seminomatous group. After comparative analysis between the experimental groups, a total of 1023 proteins were quantified and 258 were differentially expressed (Figure 1). More than half (58%) of the DEPs were overexpressed (149 proteins), while 20% were underexpressed (51 proteins) in the non-seminomatous group. Furthermore, 10% were unique to seminomatous group (25 proteins) and 12% unique to non-seminomatous group (33 proteins) (Figure 1).

### 2.3. Selection of Key DEPs for Validation

According to the Ingenuity Pathway Analysis (IPA), the category with the highest *p*-value range (1.21 × 10^−5^ − 9.72 × 10^−20^) within the top diseases and bio functions related to “physiological system development and function” was “reproductive system development and function,” which included 19 proteins. From these proteins, we selected five proteins involved in specific reproductive processes (Table 2): acrosin precursor (ACR), T-complex protein 1 subunit gamma (CCT3), and proteasome activator complex subunit 4 (PSME4). Moreover, we selected the chaperonin containing TCP1 subunit 6B (CCT6B) and S100 calcium-binding protein A9 (S100A9) for further validation as the former is reportedly involved in the cytoskeleton assembly during the spermatogenesis [23] and the latter in the cellular response to a different kind of stress [24]. The subcellular location, abundance and expression pattern of the five selected proteins obtained by the proteomic analysis is presented in Table 3. All the selected proteins were overexpressed in the group of patients with non-seminomatous TGCTs relative to those with seminomatous TGCTs, except S100A9 that were underexpressed.

### 2.4. Western Blotting

**Figure 2:**
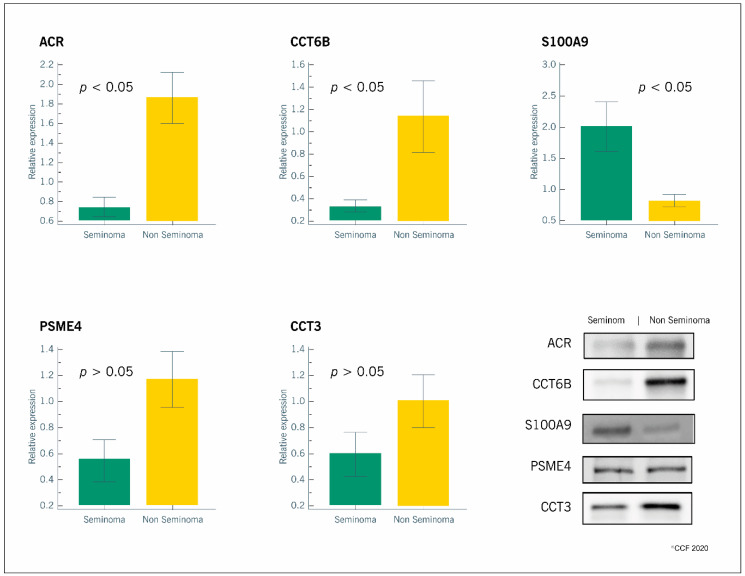
Graphical representation of the expression levels of proteins involved in reproductive functions (ACR, CCT6B, S100A9, PSME4 and CCT3) in spermatozoa samples obtained from patients with seminomatous (*n* = 6) or non-seminomatous (*n* = 6) testicular germ cell tumors. Results are presented as relative expression (mean ± SEM). Significantly different results between the two groups are indicated as *p* < 0.05. Representative blots for each protein are also presented.

All the selected proteins were identified by WB. We confirmed the proteomic results for the overexpressed (ACR, CCT6B, CCT3, PSME4) and the underexpressed (S100A9) proteins in the non-seminomatous group relative to the seminomatous group (Figure 2).

## 3. Discussion

The decline in male reproductive health over the last decades is evidenced by the decreasing male fertility rates [25]. The causes for this scenario are still poorly understood and many infertility cases (up to 30%) lacking a definitive diagnosis are categorized as “idiopathic” [26]. Infertile men have an increased risk of developing TGCTs compared to fertile men [27], and most of TGCTs are diagnosed in patients seeking for medical assistance when trying to have children and not as a routine procedure when evaluating a man’s fertility status. This is partly because the common assessment of TGCTs is based on the histological analysis of a biopsy from the testicular mass [14]. In this scenario, the identification of a sperm biomarker for a non-invasive and early diagnosis of TGCTs is of great interest as well as a way to include the screening for testicular cancer in a routine fertility evaluation. Although there is a lack of studies in this field, especially due to the difficulty in obtaining samples from these patients, sperm proteomics has emerged as an excellent approach to investigate the pathophysiology of male reproductive disorders [19,20] and as an adjuvant to fertility diagnostic testing [28]. Previous high-throughput proteomic analysis from our group identified the altered expression levels of several sperm proteins in patients with seminomatous TGCTs [21] and non-seminomatous TGCTs [22] relative to fertile men. In the present study, we compared the sperm proteome of patients with seminomatous and non-seminomatous TGCTs and attempted to identify protein biomarkers that could be used to distinguish these tumors.

Semen parameters of patients with seminomatous or non-seminomatous TGCTs were examined before initiating the cancer treatment and before cryopreservation of the samples. There were no differences in the semen parameters among these patients. It was previously reported that the sperm quality of patients with TGCTs is significantly lower relative to proven fertile men [21,22]. However, in our study the semen parameters were within the normal standards defined by the WHO, the analysis of semen parameters per se is not enough to define the fertility status of a man [29]. In fact, many infertile men present normal sperm parameters [30].

The validation of selected sperm proteins in the non-seminomatous group further confirmed that ACR and CCT6B were overexpressed while S100A9 was underexpressed. These data highlight a potential for these proteins to serve as biomarkers for the diagnosis of TGCTs, but still we have to dissect the biological significance of the results. During acrosome reaction, the inactive form of ACR (proacrosin) is converted to its active form (acrosin), which plays a key role in sperm binding to the oocyte [31]. The overexpression of proacrosin in the spermatozoa of patients with non-seminomatous TGCTs relative to those with seminomatous TGCTs suggests a premature acrosome reaction and/or impaired sperm-oocyte binding, which hampers male fertility potential. This may also be associated to cytoskeleton dynamics instability since CCT6B was also found to be overexpressed in patients with non-seminomatous TGCTs. CCT6B is a cytosolic subunit of the chaperonin-containing T-complex (TRiC), a larger chaperone complex mediating the folding of cytoskeleton proteins, such as actin and tubulin, by means of ATP hydrolysis [23]. In addition CCT6B was overexpressed in idiopathic infertile patients after antioxidant treatment [32] highlighting its relevance for male fertility. As the major function of this protein is to support cytoskeleton organization, and that is pivotal for spermatogenesis and oocyte binding, this data further supports that both functions are primary triggers for TGCTs. CCT6B was also previously described in other scenarios, such as Burkitt lymphoma: nonsense and frame shift gene mutations were suggested to cause a protein loss of function, although its role in this disease needed to be further investigated [33]. Moreover, a reduced synthesis of CCT6B mRNA was observed in hepatocellular carcinoma when compared to healthy tissue while the other components of TRiC complex were overexpressed [34]. The upregulation of several TRiC subunits were previously associated with high proliferative cancer rate [35,36]. Thus, it is possible that CCT6B overexpression in the non-seminomatous testicular cancer group may explain the higher invasiveness capacity of non-seminomatous TGCTs relative to seminomatous TGCTs. Further studies will be necessary to explore this hypothesis and if CCT6B expression in spermatozoa of males can serve as a biomarker for TGCTs onset and progression.

Interestingly, we also validated the underexpression of S100A9 in spermatozoa of non-seminomatous TGCTs patients. S100A9 is a Ca^2+^/Zn^2+^ binding protein involved in the start of the inflammatory response to cellular stress [24]. It stimulates the neutrophils chemotaxis on the site of inflammation and can enhance their phagocytic activity [37,38]. We have previously reported an overexpression of S100A9 in spermatozoa of patients with high levels of oxidative stress and we suggested its involvement in the activation of pro-inflammatory cytokines [39]. Another study showed an overexpression of S100A9 in patients with seminomatous TGCTs relative to fertile men [21]. The observed underexpression of S100A9 in patients with non-seminomatous TGCTs when compared to those with seminomatous TGCTs may be due to the different cellular response related to different types of cancer. In addition, non-seminomatous cancer is associated to reduced cellular ability to induce an inflammatory status, which may explain the higher invasiveness and the worse prognosis.

We also selected for validation the protein PSME4 due to its role in chromatin remodeling and DNA double-strand break repair during spermatogenesis [40]. Although it was identified as overexpressed in the group of non-seminomatous TGCTs by the proteomic analysis, we did not observe any differences when validating by WB. The same was observed for the protein CCT3, which is one of the subunits of the TRiC complex involved in the binding of capacitated spermatozoa to the zona pellucida [41]. In a previous proteomic study using the same antibodies for PSME4 and CCT3, we also failed to validate the alterations in these proteins by WB, while the proteomic data clearly showed that they were differentially expressed between the study groups [21]. Currently, the validation of proteomic results by WB is a popular matter of debate. Although it is robust to affirm that alterations detected by this technique clearly reflect changes in the proteomic profile, it has some major limitations including: (1) the user has to select the proteins with high abundance in the proteomic analysis to increase the probability of validation by WB, which has a lower sensitivity; and (2) the use of housekeeping proteins as internal standard for WB analysis, because their expression may be different in health or disease conditions [42]. We tried to overcome the above limitations by applying the following criteria: (1) selection of proteins with very low, low, medium, and high abundance; and (2) use of total protein staining as internal standard for WB analysis rather than the expression of a housekeeping protein. However, WB is characterized by a lower specificity and sensitivity than proteomics analysis in terms of proteins identification, mainly due to the detection of a chemiluminescence signal and the further densitometric analysis. On the other side, LC-MS/MS automatically identifies a protein starting from two single peptide fragments. WB is a valid tool to validate and/or strengthens proteomic results, but it cannot match the sophisticated high throughput proteomics approach for the investigation of DEPs in clinical scenarios.

In conclusion, our results highlight that there is a distinct proteomic profile in the spermatozoa from patients with seminomatous and those with non-seminomatous TGCTs. We validated by WB the overexpression of ACR and CCT6B as well as the underexpression of S100A9 in the spermatozoa of patients with non-seminomatous TGCTs, which was previously identified by the LC-MS/MS analysis. Although WB analysis failed to confirm proteomics data for CCT3 and PSME4, our overall results suggest an association between the higher and faster invasiveness of non-seminomatous TGCTs and the altered protein expressions, providing important information for future studies. Analyzing the expression of certain sperm proteins as a routine procedure during fertility testing can be a useful tool to detect the onset and progression of diseases such as TGCTs.

## 4. Materials and Methods

### 4.1. Study Design

The present study used semen samples from patients with seminomatous or non-seminomatous TGCTs (*n* = 15/group). The diagnosis was confirmed by a physician after histological analysis of a biopsy of the testicular mass according to the WHO criteria [14]. The semen samples were cryopreserved before the start of patients’ cancer therapy and were only used after patient’s consent for disposal of their banked specimen. This study is a continuation of our previous published studies [21,22] and the participants are the same. The study design was reviewed and approved by the Institutional Review Board (IRB) of the Cleveland Clinic (IRB #13-1554) on the 24th of December 2013. An informed written consent was signed by all the patients enrolled in the study.

### 4.2. Collection and Storage of Samples

Semen samples were collected at the Andrology Center, Cleveland Clinic, after 2–3 days of abstinence. Liquefaction occurred for 20–30 min at 37 °C and a routine semen analysis was conducted according to the WHO 2010 guidelines [43]. The TEST-yolk buffer (TYB; Irvine Scientific, Santa Ana, CA, USA) was used to cryopreserve the semen samples in a ratio 1:1, as previously described [44].

### 4.3. Total Protein Extraction

The cryopreserved semen samples were thawed on ice and then centrifuged at 4000 *g* for 10 min. In order to remove the cryoprotectant, the sperm pellets were washed four times in phosphate-buffered saline (PBS; Irvine Scientific, Santa Ana, CA, USA) by repeated centrifugations at 4000 *g* for 10 min at 4 °C. Radio-immunoprecipitation assay (RIPA; Sigma-Aldrich, St. Louis, MO, USA) buffer supplemented with Protease Inhibitor Cocktail (cOmplete^TM^ ULTRA Tablets, EDTA-free, Roche, Mannheim, Germany) was added to each sperm pellet (100 µL RIPA/10^6^ sperm) and incubated overnight at 4 °C to allow cell lysis. Samples were then centrifuged at 10,000 g for 30 min at 4 °C to recover the protein fraction (supernatant). Protein estimation was performed by Pierce BCA Protein Assay kit (Thermo Fisher Scientific, Waltham, MA, USA) according to the manufacturer’s instructions.

### 4.4. Shotgun Proteomic Analysis

Three protein samples from seminomatous or non-seminomatous groups were randomly selected for the proteomic analysis by liquid chromatography-tandem mass spectrometry (LC-MS/MS). Samples were pooled (*n* = 3) using the same amount of protein from each sample and each pool was assessed as an individual sample. A Finnigan LTQ-Orbitrap Elite hybrid mass spectrometer (Thermo Fisher Scientific, Waltham, MA, USA) was used as previously described [45,46]. Scaffold (Proteome Software Inc., Portland, OR, USA; version 4.0.6.1) was used for the identification of the differentially expressed proteins (DEPs) between the seminomatous and non-seminomatous groups. The spectral counts were used to determine the abundance of each protein (very low, low, medium or high). The expression profile of the identified DEPs (unique, underexpressed, or overexpressed) was based on the normalized spectral abundance factor (NSAF) ratio (Supplementary Table S1). Proteomic analysis was conducted in compliance with the Minimum Information about a Proteomics Experiment (MIAPE) guidelines of the Human Proteome Organization’s Proteomics Standards Initiative (HUPO-PSI) [47].

### 4.5. Bioinformatic Analysis

Bioinformatic analysis of the identified DEPs was conducted by the IPA (Qiagen, Hilden, Germany) software. IPA was used to evaluate the diseases and bio functions, canonical pathways, and cellular sublocation related to the identified DEPs. The bioinformatic criteria to select DEPs for further validation by WB were: (1) proteins involved in the “reproductive system development and function”; (2) proteins involved in the top canonical pathways; (3) proteins related to sperm function that are well-described in the literature.

### 4.6. Western Blotting

Western blotting (WB) was performed using individual samples from the seminomatous (*n* = 6) and non-seminomatous (*n* = 6) groups. A total of 25 µg/sample was mixed with 4× Laemmli sample buffer (Bio-Rad, Hercules, CA, USA) in a ratio 1:3 and completed up to 25 µL with PBS. Samples were boiled at 95 °C for 10 min and immediately loaded into a 4–15% (*w*/*v*) polyacrylamide gel (Bio-Rad, Hercules, CA, USA). Electrophoresis was performed with constant voltage (90 V) for 2 h. Precision Plus Protein™ Dual Xtra Standards (Thermo Fisher Scientific, Waltham, MA, USA) was used as the molecular weight marker. Proteins were then transferred (18 V for 30 min) to methanol-activated polyvinylidene difluoride (PVDF) membranes (GE Healthcare, Marlborough, MA, USA) and blocked for 90 min at room temperature, with a 5% (*w*/*v*) non-fat milk (Bio-Rad, Hercules, CA, USA) solution prepared in tris-buffered saline with tween-20 (TBST; Sigma-Aldrich, St. Louis, MO, USA). Membranes were incubated overnight (4 °C) with specific primary antibodies followed by the respective secondary antibodies at room temperature, for 90 min (Supplementary Table S2). Membranes were reacted with enhanced chemiluminescence (ECL) reagent (GE Healthcare, Marlborough, MA, USA) for 5 min and read with the ChemiDoc™ MP Imaging System (Bio-Rad, Hercules, CA, USA) to detect the chemiluminescence signal. Densities from each band were obtained with Image Lab^TM^ Software (Bio-Rad, Hercules, CA, USA) according to standard methods and divided by the corresponding total protein lane density. Total protein density was obtained by incubation of the membranes with total colloidal gold protein stain (Bio-Rad, Hercules, CA, USA). Results were expressed as relative expression.

### 4.7. Statistical Analysis

The Grubbs’ test was performed to identify possible outliers and the Kolmogorov-Smirnov test was used to check if the data followed a normal distribution. As our data did not follow a normal distribution, semen parameters and WB results were analyzed by the non-parametric Mann-Whitney test for independent samples, using the MedCalc Software (V. 17.8; MedCalc Software, Ostend, Belgium). All data are presented as mean ± SEM and differences with *p* < 0.05 were considered statistically significant.

## Figures and Tables

**Table 1:** Semen parameters of patients (*n* = 15 per group) with seminomatous and non-seminomatous testicular germ cell tumors.

Parameter	Seminomatous	Non-Seminomatous	*p*-Value
Semen volume (mL)	3.33 ± 0.42	3.67 ± 0.59	0.8990
Sperm motility (%)	54 ± 5	59 ± 7	0.2628
Sperm concentration (10^6^/mL)	46.72 ± 12.19	48.71 ± 17.12	0.9835
Total sperm count (10^6^)	136.11 ± 41.55	166.12 ± 56.17	0.7875
Total motile count (10^6^)	75.63 ± 22.44	108.36 ± 35.95	0.7557

Results are presented as mean ± SEM. Results were considered statistically significant for *p* < 0.05.

**Table 2:** Specific diseases and bio functions of the differentially expressed proteins (DEPs) selected for validation by western blotting.

Process	Protein	*p*-Value
Binding of sperm	ACR, CCT3	9.72 × 10^−20^
Cell death	CCT3, S100A9	1.73 × 10^−19^
Necrosis	CCT3	1.70 × 10^−19^
Binding of zona pellucida	ACR, CCT3	1.56 × 10^−19^
Cancer	CCT3, CCT6B	8.22 × 10^−12^
Tumorigenesis of tissue	ACR, CCT3, PSME4, CCT6B	1.80 × 10^−9^
Apoptosis	PSME4, S100A9	1.27 × 10^−9^
Asthenozoospermia	ACR, PSME4	2.79 × 10^−5^
Malignant neoplasm of male genital organ	PSME4	2.13 × 10^−5^
Acrosome reaction	ACR	1.21 × 10^−5^

Abbreviations: ACR, acrosin precursor; CCT3, T-complex protein 1 subunit gamma; HSPA2, heat shock-related 70 kDa protein 2; PSME4, proteasome activator complex subunit 4; CCT6B, chaperonin containing TCP1 subunit 6B; S100A9, S100 calcium-binding protein A9.

**Table 3:** Proteomic data of the differentially expressed proteins (DEPs) identified in the spermatozoa samples of patients with seminomatous and non-seminomatous testicular germ cell tumors before cancer therapy, which were selected for validation by western blotting.

Protein	Subcellular Location	Abundance		NSAF Ratio	Expression Profile	*p*-Value
		Seminomatous	Non-Seminomatous			
**ACR**	Extracellular space	Medium	High	1.77	OE in Non-seminomatous	0.0005
**CCT3**	Cytoplasm	Very Low	Medium	9.24	OE in Non-seminomatous	<0.0001
**PSME4**	Cytoplasm	Very Low	Medium	4.81	OE in Non-seminomatous	0.0023
**CCT6B**	Cytoplasm	Very Low	Low	3.12	OE in Non-seminomatous	0.0021
**S100A9**	Cytoplasm	Medium	Low	0.32	UE in Non-seminomatous	0.0005

Abbreviations: ACR, acrosin precursor; CCT3, T-complex protein 1 subunit gamma; PSME4, proteasome activator complex subunit 4; CCT6B, chaperonin containing TCP1 subunit 6B; S100A9, S100 calcium-binding protein A9; OE, overexpressed; UE, underexpressed.

## Supplementary Materials

Supplementary materials can be found at https://www.mdpi.com/1422-0067/21/14/4817/s1.
