# Supplementary material for: Distinct Proteomic Profile of Spermatozoa from Men with Seminomatous and Non-Seminomatous Testicular Germ Cell Tumors
Source: Int J Mol Sci. 2020 Jul 8;21(14):4817. doi: 10.3390/ijms21144817 (PMC7404221; doi:10.3390/ijms21144817)
Supplement: Supplementary file 1 [file ijms-21-04817-s001.zip › Supplementary Files/Supplementary Table 1.docx]

**Supplementary Table 1.** Criteria used to determine if proteins are differentially expressed based on spectral count.

| **Abundance** | **SC range** | **NSAF ratio** | ***P*-value** |
| --- | --- | --- | --- |
| Very Low (VL) | 1.7 – 7 | ≤ 0.4 – underexpressed; ≥ 2.5 – overexpressed | ≤ 0.001 |
| Low (L) | 8 – 19 | ≤ 0.4 – underexpressed; ≥ 2.5 – overexpressed | ≤ 0.01 |
| Medium (M) | 20 – 79 | ≤ 0.5 – underexpressed; ≥ 2.0 – overexpressed | ≤ 0.05 |
| High (H) | ≥80 | ≤ 0.67 – underexpressed; ≥ 1.5 – overexpressed | ≤ 0.05 |

Abbreviations: SC, spectral count; NSAF, normalized spectral abundance factor.
