# Supplementary material for: Distinct Proteomic Profile of Spermatozoa from Men with Seminomatous and Non-Seminomatous Testicular Germ Cell Tumors
Source: Int J Mol Sci. 2020 Jul 8;21(14):4817. doi: 10.3390/ijms21144817 (PMC7404221; doi:10.3390/ijms21144817)
Supplement: Supplementary file 1 [file ijms-21-04817-s001.zip › Supplementary Files/Supplementary Table 2.docx]

**Supplementary Table 2.** List of the primary and secondary antibodies used.

| Antibody | Source | Dilution | Vendor | Catalog # |
| --- | --- | --- | --- | --- |
| ACR | Rabbit | 1:1000 | Abcam | ab203289 |
| CCT3 | Rabbit | 1:2000 | Abcam | ab225878 |
| PSME4 | Rabbit | 1:500 | Abcam | ab181203 |
| CCTB6 | Rabbit | 1:1000 |  | ab168299 |
| S100A9 | Rabbit | 1:1000 |  | ab92507 |
| Mouse* | Rabbit | 1:10000 | Abcam | ab6728 |
| Rabbit* | Goat | 1:10000 | Abcam | ab97051 |

Abbreviations: ACR, acrosin precursor; CCT3, T-complex protein 1 subunit gamma; PSME4, proteasome activator complex subunit 4; CCT6B, chaperonin containing TCP1 subunit 6B; S100A9, S100 calcium binding protein A9.

* Secondary antibody.
